# Diagnostic value of [^18^F]FDG PET/MRI for staging in patients with ovarian cancer

**DOI:** 10.1186/s13550-020-00712-3

**Published:** 2020-10-02

**Authors:** Hideaki Tsuyoshi, Tetsuya Tsujikawa, Shizuka Yamada, Hidehiko Okazawa, Yoshio Yoshida

**Affiliations:** 1 Department of Obstetrics and Gynecology, University of Fukui, 23-3 Matsuoka-Shimoaizuki, Eiheiji-cho, Yoshida-gun, Fukui 910-1193 Japan; 2 Biomedical Imaging Research Center, University of Fukui, Fukui, Japan

**Keywords:** [^18^F]FDG PET/MRI, Contrast-enhanced CT, Contrast-enhanced MRI, Ovarian cancer

## Abstract

**Purpose:**

To evaluate the diagnostic potential of PET/MRI with 2-[^18^F]fluoro-2-deoxy-d-glucose ([^18^F]FDG) in ovarian cancer.

**Materials and methods:**

Participants comprised 103 patients with suspected ovarian cancer underwent pretreatment [^18^F]FDG PET/MRI, contrast-enhanced CT (ceCT) and pelvic dynamic contrast-enhanced MRI (ceMRI). Diagnostic performance of [^18^F]FDG PET/MRI and ceMRI for assessing the characterization and the extent of the primary tumor (T stage) and [^18^F]FDG PET/MRI and ceCT for assessing nodal (N stage) and distant (M stage) metastases was evaluated by two experienced readers. Histopathological and follow-up imaging results were used as the gold standard. The McNemar test was employed for statistical analysis.

**Results:**

Accuracy for the characterization of suspected ovarian cancer was significantly better for [^18^F]FDG PET/MRI (92.5%) [95% confidence interval (CI) 0.84–0.95] than for ceMRI (80.6%) (95% CI 0.72–0.83) (*p* < 0.05). Accuracy for T status was 96.4% (95% CI 0.96–0.96) and 92.9% (95% CI 0.93–0.93) for [^18^F]FDG PET/MRI and ceMRI/ceCT, respectively. Patient-based accuracies for N and M status were 100% (95% CI 0.88–1.00) and 100% (95% CI 0.88–1.00) for [^18^F]FDG PET/MRI and 85.2% (95% CI 0.76–0.85) and 30.8% (95% CI 0.19–0.31) for ceCT and M staging representing significant differences (*p* < 0.01). Lesion-based sensitivity, specificity and accuracy for N status were 78.6% (95% CI 0.57–0.91), 95.7% (95% CI 0.93–0.97) and 93.9% (95% CI 0.89–0.97) for [^18^F]FDG PET/MRI and 42.9% (95% CI 0.24–0.58), 96.6% (95% CI 0.94–0.98) and 90.8% (95% CI 0.87–0.94) for ceCT.

**Conclusions:**

[^18^F]FDG PET/MRI offers better sensitivity and specificity for the characterization and M staging than ceMRI and ceCT, and diagnostic value for T and N staging equivalent to ceMRI and ceCT, suggesting that [^18^F]FDG PET/MRI might represent a useful diagnostic alternative to conventional imaging modalities in ovarian cancer.

## Background

Ovarian cancer is the most lethal gynecological malignancy, ranking as the fifth-most common cause of cancer death among women. The standard treatment is debulking surgery followed by 6 cycles of chemotherapy. In presumed early ovarian cancer, staging laparotomy including hysterectomy, bilateral salpingo-oophorectomy, pelvic and para-aortic lymphadenectomy and omentectomy is performed to stage the disease based on the International Federation of Gynecology and Obstetrics (FIGO) and/or Union for International Cancer Control (UICC) TNM classifications [1]. For patients with advanced disease in which complete debulking is not feasible, neoadjuvant chemotherapy (NAC) followed by interval debulking surgery (IDS) may also be acceptable [2]. Accurate preoperative assessment including the differentiation of benign and malignant disease or the diagnosis of nodal, peritoneal or distant disease is necessary for optimal treatment planning.

To characterize ovarian tumors as benign or malignant, magnetic resonance imaging (MRI) with intravenous administration of contrast provides the highest post-test probability of detecting ovarian cancer when compared with computed tomography (CT), Doppler ultrasonography (US) or MRI without contrast administration [3–5]. Positron emission tomography (PET), particularly 2-[^18^F]fluoro-2-deoxy-d-glucose ([^18^F]FDG) as a tracer reflecting cellular metabolism, has been shown to be worth consideration alongside conventional imaging modalities. For the detection of lymph node metastasis, distant metastasis, peritoneal disease or recurrent disease in ovarian cancer, [^18^F]FDG PET/CT could be useful compared with conventional modalities including CT or MRI [6–10]. However, [^18^F]FDG PET/CT has a limited and controversial role to play in the characterization of ovarian tumors, because the physiologically increased uptake of FDG into the normal ovaries leads to false-positive results or low diagnostic value in differentiating between borderline and benign tumors due to low FDG uptake leading to false-negative results [11].

The new PET modality of [^18^F]FDG PET/MRI provides high soft-tissue contrast along with functional imaging of FDG uptake and has shown potentially better diagnostic performance than [^18^F]FDG PET/CT in gynecologic cancers [12, 13]. In evaluating and characterizing ovarian tumors, fusion of PET and MRI provides advantageous sensitivity and specificity compared with MRI or [^18^F]FDG PET/CT [14]. Moreover, in the assessment of tumor resectability at PDS among patients with advanced ovarian cancer, MRI or [^18^F]FDG PET/CT could provide high specificity and moderate sensitivity [15], suggesting that integrated PET/MRI combining the individual advantages of PET and MRI may have a role to play in the characterization of ovarian tumors or the pretreatment evaluation of ovarian cancer, while integrated PET/MRI has not yet been well studied in ovarian cancer.

The aim of our study was thus to evaluate the diagnostic utility of integrated [^18^F]FDG PET/MRI for the characterization, whole-body tumor staging and restaging of patients with ovarian cancer, and to compare the diagnostic accuracy of integrated [^18^F]FDG PET/MRI with that of contrast-enhanced CT (ceCT) or contrast-enhanced MRI (ceMRI).

## Materials and methods

### Patients

We retrospectively reviewed the medical records of 135 patients with suspected ovarian cancer or recurrence between February 2016 and May 2019 (Additional file 1: Table S1). Of these, 103 patients (mean age, 55.5 years; age range, 11–80 years) who had undergone [^18^F]FDG PET/MRI, ceCT and pelvic dynamic ceMRI with obtained informed consent for the characterization, initial staging and determination of the presence of residual disease after NAC and detection of recurrence based on the Japanese Imaging Guideline from Japan Radiological Society were included in the present study. Patients had completed [^18^F]FDG PET/MRI, ceCT and ceMRI within 4 months (mean, 29.1 days; range, 1–103 days) prior to treatment. The maximum interval among [^18^F]FDG PET/MRI, ceCT and ceMRI was 121 days (mean, 14.3 days; range, 0–121 days). Of the 103 patients, 67 patients with suspected ovarian cancer were characterized using [^18^F]FDG PET/MRI and ceMRI. The 56 patients with pathologically or cytologically proven diagnoses of ovarian cancer underwent initial staging with [^18^F]FDG PET/MRI, ceCT and ceMRI. Seven patients with pathologically or cytologically confirmed diagnoses of ovarian cancer were evaluated for the presence of residual disease after NAC and 11 with pathologically proven diagnosis of ovarian cancer were evaluated for recurrence with [^18^F]FDG PET/MRI and ceCT. This was a multi-center study, as 54 patients with data from ceCT and/or ceMRI were referred from other institutions, although all patients underwent [^18^F]FDG PET/MRI in our institution.

### [^18^F]FDG PET/MRI

#### Whole-body PET/MRI

Patients fasted for at least 4 h prior to intravenous injection of 200 MBq of [^18^F]FDG. Fifty minutes after injection, patients were transferred to a whole-body 3.0-T PET/MR scanner (Signa PET/MR; GE Healthcare, Waukesha, WI). Anatomical coverage was from the vertex to the mid-thigh. PET acquisition was performed in 3-dimensional (3D) mode with 5.5 min/bed position (89 slices/bed) in 5–6 beds with a 24-slice overlap. A 2-point Dixon 3D volumetric interpolated T1-weighted fast spoiled gradient echo sequence was acquired at each table position and was used to generate MR attenuation correction (MR-AC) maps. Dixon-based MR-AC classifies body tissues into soft tissue, fat and air. PET data were reconstructed by ordered subset expectation maximization (OSEM), selecting 14 subsets and 3 iterations, and post-smoothing with a 3-mm Gaussian filter. Reconstructed images were then converted to semiquantitative images corrected by the injected dose and the body weight of the subject as the standardized uptake value (SUV).

#### Pelvic PET/MRI

After whole-body scanning and a brief break for urination, the patient was repositioned in the PET/MR scanner. The pelvic PET scan was performed as a 3D acquisition in list mode with 15 min/bed position (89 slices/bed) in 1–2 beds with a 24-slice overlap. Regional PET data were reconstructed with OSEM selecting 16 subsets and 4 iterations, and post-smoothing with a 4-mm Gaussian filter. Reconstructed images were then converted to SUV images. For pelvic MRI, T2-weighted images were acquired in the sagittal, transaxial and coronal planes, using the following T2-weighted image parameters: TR, 4000–7000 ms; TE, 146 ms; section thickness, 4 mm; section overlap, 0 mm; flip angle, 100°; FOV, 240 × 240 mm; matrix, 384 × 384; two excitations; and bandwidth, 83.3 kHz.

### Dynamic contrast-enhanced (DCE) MRI

Pelvic MRI was performed using a 3-T clinical scanner (Discovery MR750; GE Healthcare, Waukesha, WI) in 27 patients. To delineate the anatomy of the pelvis prior to pelvic DCE-MRI, T2-weighted imaging was performed in the sagittal, transaxial and coronal planes. The following T2-weighted image parameters were used: TR, 3200–6000 ms; TE, 60–85 ms; section thickness, 4 mm; interval, 1 mm; flip angle, 111°; FOV, 240 × 240 mm; matrix, 320 × 224; two excitations; echo train length, 10; and bandwidth, 62.5 kHz. For DCE-MRI, a sagittal 3D fast spoiled-gradient-recalled T1-weighted sequence using the Dixon method with fat suppression (LAVA Flex; GE Healthcare) was used with the following parameters: TR, 5.0 ms; TE, 1.3 ms; section thickness, 3 mm; flip angle, 12°; FOV, 260 × 260 mm; matrix, 320 × 192; 1 excitation; and bandwidth, 166.7 kHz. After non-contrast images were acquired, 0.2 ml/kg of gadolinium-based contrast agent was injected at a rate of 2 ml/s using a contrast injector, followed by a 20-ml saline flush. Image sets were acquired at multiple phases, at 45, 80 and 120 s after initiation of injection. In 40 patients, DCE-MRI was performed at other institutes using 1.5-T clinical scanners (Magnetom Aera; Siemens Healthineers, or Signa HDe; GE Healthcare).

### ceCT

CT examinations covering the chest, abdomen and pelvis were performed using a 64-slice multidetector CT scanner (Discovery CT 750HD; GE Medical Systems, Milwaukee, WI) before and after intravenous administration of nonionic iodinated contrast material (iopamidol, Iopamiron 300; Schering, Berlin, Germany).

### Image interpretation

Images were analyzed on a dedicated workstation (Advantage Workstation 4.6; GE). Two board-certificated radiologists/nuclear medicine physicians, each with double certifications and specializing in gynecological imaging, evaluated the [^18^F]FDG PET/MRI, ceCT and ceMRI images retrospectively and reached consensus decisions. Images were evaluated for the following: (a) characterization; (b) tumor extension into the uterus, fallopian tubes or ovaries (T2a); (c) tumor extension into other nearby pelvic organs such as the bladder, sigmoid colon or rectum (T2b); (d) tumor extension into organs outside the pelvis, no bigger than 2 cm in extent (T3b); (e) tumor extension into organs outside the pelvis, larger than 2 cm in extent (T3c); (f) pelvic or para-aortic lymph nodes (N); (g) distant metastasis (M); (h) residual disease for IDS after NAC; and (i) recurrence. The present study applied the TNM classification to evaluate the diagnostic value of the imaging modalities, because this anatomically based system separately records the primary and regional nodal extent of the tumor and the absence or presence of metastases. Diagnostic performance of [^18^F]FDG PET/MRI and ceMRI for assessing the characterization and extent of the primary tumor and [^18^F]FDG PET/MRI and ceCT for assessing nodal and distant metastases was evaluated. Both readers were blinded to the results of other imaging studies, histopathologic findings and clinical data. Each dataset was reviewed as the consensus decisions of the two readers after a minimum interval of three weeks to avoid any decision threshold bias due to reading-order effects. For CT and MRI interpretation, several previous standard criteria related to primary tumor and nodal or distant metastatic staging of ovarian cancer were used as the reference criteria [16]. Swollen lymph nodes larger than 1 cm in short-axis diameter were graded as malignant. For [^18^F]FDG PET/MRI interpretations, the classification of lymph nodes as cancer-positive was based on the presence of focally appreciable metabolic activity above that of normal muscle; or asymmetric metabolic activity greater than that of normal-appearing lymph nodes at the same level in the contralateral pelvis, in a location corresponding to the lymph node chains on CT or MRI images, with reference to previous reports [12, 13]. Furthermore, the presence of a central unenhanced area suggesting central necrosis or peripheral low attenuation suggesting a fatty hilum within lymph nodes was considered a benign sign. Tumor invasion of neighboring structures was decided primarily on the basis of CT or MRI findings, with reference to the [^18^F]FDG PET findings.

### Reference standard

Histopathological results were used as the standard of reference for the characterization, T, N and M staging, determination of residual disease after NAC and determination of recurrence. Because clinical and ethical standards of patient management do not require surgery or sampling of all detected lesions, a modified reference standard was used for lesions without histopathological sampling to take into account all prior and follow-up imaging. A decrease in size and/or SUVmax under therapy or an increase in size and/or SUVmax without therapy was regarded as a sign of malignancy. PET-negative and inconspicuous lesions with constant size were rated as benign.

### Statistical analysis

The McNemar test was used to determine the statistical significance of differences in the accuracy of T, N and M staging as determined by PET/MRI, ceCT and ceMRI. Statistical analysis was performed using PRISM version 6.0 software (GraphPad, San Diego, CA). Differences at the level of *p* < 0.05 were considered statistically significant.

## Results

### Patients

According to the revised FIGO criteria [1], T stage was classified as pT1 in 30 patients, pT2a in three, pT2b in one, pT3b in one and pT3c in 21. The histopathologic types of primary tumors with malignancy or borderline malignancy were high-grade serous carcinoma (*n* = 16), low-grade serous carcinoma (*n* = 1), serous borderline tumor (*n* = 2), adenocarcinoma proven from ascites or pleural effusion (*n* = 2), mucinous carcinoma (*n* = 1), mucinous borderline tumor (*n* = 5), seromucinous carcinoma (*n* = 1), seromucinous borderline tumor (*n* = 2), endometrioid carcinoma (*n* = 6), carcinosarcoma (*n* = 2), clear cell carcinoma (*n* = 7), undifferentiated carcinoma (*n* = 2), Sertoli–Leydig cell tumor (poorly differentiated (*n* = 1), moderately differentiated (*n* = 1)), immature teratoma grade 1 (*n* = 4), squamous cell carcinoma (*n* = 2) and large cell neuroendocrine carcinoma (*n* = 1). N stage was classified as N0 in 50 patients, and N1 in six including pelvic and/or para-aorta lymph nodes. M stage was classified as M0 in 44 patients and M1 in 12 involving liver and extra-abdominal lymph nodes including sternal, supraclavicular, subclavicular, axillary and longitudinal lymph nodes. Demographic data for the 56 patients are listed in Table 1. Histopathologic types of primary benign tumors with suspected malignancy were endometrial cyst (*n* = 7), mucinous cystadenoma *n* = 7), mature cystic teratoma (*n* = 5), serous cystadenoma (*n* = 4), struma ovalii (*n* = 1), fibroma (*n* = 1), thecoma (*n* = 1), lymphangioma (*n* = 1) and abscess (*n* = 1) (Table 2). The histopathologic types of ovarian cancer after NAC were high-grade serous carcinoma (*n* = 6), and carcinosarcoma (*n* = 1) (Table 3). The histopathologic types of recurrent ovarian cancer were high-grade serous carcinoma (*n* = 5), clear cell carcinoma (*n* = 2), large cell neuroendocrine carcinoma (*n* = 2), seromucinous carcinoma (*n* = 1) and adenocarcinoma (*n* = 1) (Table 4).

**Table 1:** Characteristics of patients with primary ovarian cancer

Case	Age (years)	Histology	Pathological staging	PET/MRI staging	ceMRI and/or ceCT staging
1	40	Clear	T1N0M0	T1N0M0	T1N0M0
2	52	LGSC	T1N0M0	T1N0M0	T1N0M0
3	41	Clear	T1N0M0	T1N0M0	T1N0M0
4	47	Endometrioid	T3cN0M1	T3cN0M1	T3cN0M0
5	58	Seromucinous	T1N0M0	T1N0M0	T1N0M0
6	52	Clear	T2aN0M0	T1N0M0	T1N0M0
7	65	Mucinous borderline	T1N0M0	T1N0M0	T1N0M0
8	19	Immature teratoma, G1	T1N0M0	benign	benign
9	69	Clear	T1N0M0	T1N0M0	T1N0M0
10	42	Mucinous borderline	T1N0M0	T1N0M0	T1N0M0
11	45	HGSC	T2bN0M0	T1N0M0	T1N0M0
12	76	Mucinous borderline	T1N0M0	T1N0M0	T1N0M0
13	73	Sertoli–Leydig moderate	T1N0M0	T1N0M0	T1N0M0
14	31	Seromucinous borderline	T1N0M0	T1N0M0	T1N0M0
15	67	CS	T2aN1M1	T2aN1M1	T2aN1M0
16	77	HGSC	T3cN1M1	T3cN1M1	T3cN1M0
17	78	HGSC	T3cN0M0	T3cN0M0	T3cN0M0
18	65	Endometrioid	T3cN0M0	T3cN0M0	T3cN0M0
19	56	Endometrioid	T2aN1M0	T2aN1M0	T2aN1M0
20	78	HGSC	T3cN0M0	T3cN0M0	T3cN0M0
21	66	Clear	T1N0M0	T1N0M0	T1N0M0
22	43	Sertoli–Leydig poor	T1N0M0	T1N0M0	T1N0M0
23	20	Immature teratoma, G1	T1N0M0	T1N0M0	T1N0M0
24	62	CS	T3cN0M1	T3cN0M1	T3cN0M1
25	50	Mucinous borderline	T1N0M0	T1N0M0	T1N0M0
26	41	Serous borderline	T1N0M0	T1N0M0	T1N0M0
27	67	SCC	T1N1M1	T1N1M1	T1N0M0
28	63	HGSC	T3cN0M0	T3cN0M0	T3cN0M0
29	54	Endometrioid	T1N0M0	T1N0M0	T1N0M0
30	71	HGSC	T3cN0M1	T3cN0M1	T3cN0M0
31	73	Serous borderline	T1N0M0	benign	benign
32	73	Undifferentiated carcinoma	T3cN0M1	T3cN0M1	T3cN0M1
33	23	Immature teratoma, G1	T1N0M0	T1N0M0	T1N0M0
34	31	Neuroendocrine carcinoma	T3cN0M0	T3cN0M0	T3cN0M0
35	38	Mucinous borderline	T1N0M0	T1N0M0	T1N0M0
36	53	Seromucinous borderline	T1N0M0	T1N0M0	T1N0M0
37	43	Endometrioid	T1N0M0	T1N0M0	T1N0M0
38	61	Mucinous	T1N0M0	T1N0M0	T1N0M0
39	51	Clear	T1N0M0	T1N0M0	T1N0M0
40	62	HGSC	T3cN0M0	T3cN0M0	T3cN0M0
41	56	HGSC	T3bN0M0	T1N0M0	T1N0M0
42	68	SCC	T1N1M1	T1N1M1	T1N0M1
43	80	Undifferentiated carcinoma	T3cN1M1	T3cN1M1	T3cN1M0
44	56	Endometrioid	T1N0M0	T1N0M0	T1N0M0
45	51	Clear	T1N0M0	T1N0M0	T1N0M0
46	72	HGSC	T3cN0M0	T3cN0M0	T3cN0M0
47	75	Adenocarcinoma	T3cN0M0	T3cN0M0	T3cN0M0
48	45	HGSC	T1N0M0	T1N0M0	T1N0M0
49	11	Immature teratoma G1	T1N0M0	T1N0M0	T1N0M0
50	76	Adenocarcinoma	T3cN0M1	T3cN0M1	T3cN0M0
51	48	HGSC	T3cN0M0	T3cN0M0	T3cN0M0
52	65	HGSC	T3cN0M1	T3cN0M1	T3cN0M0
53	59	HGSC	T3cN0M0	T3cN0M0	T3cN0M0
54	66	HGSC	T3cN0M1	T3cN0M1	T3cN0M0
55	64	HGSC	T3cN0M0	T3cN0M0	T3cN0M0
56	80	HGSC	T3cN0M1	T3cN0M1	T3cN0M0

Underlining indicates over- or under-diagnosis

G, grade; HGSC, high-grade serous carcinoma; LGSC, low-grade serous carcinoma; CS, carcinosarcoma; SCC, squamous cell carcinoma

**Table 2:** Characteristics of patients with pathologically benign ovarian tumor

Characteristics	*n*	%
Total number of patients	29	
Mean age (range), years	50.7 (16–75)	
Histology		
Endometrial cyst	7	24.1
Mucinous cystadenoma	7	24.1
Mature cystic teratoma	5	17.2
Serous cystadenoma	4	13.8
Struma ovarii	2	6.9
Fibroma	1	3.4
Thecoma	1	3.4
Lymphangioma	1	3.4
Abscess	1	3.4

**Table 3:** Characteristics of patients after neoadjuvant chemotherapy

Characteristics	*n*	%
Total number of patients	7	
Mean age (range), years	67.7 (50–78)	
Histology		
HGSC	6	85.7
CS	1	14.3

HGSC, high-grade serous carcinoma; CS, carcinosarcoma

**Table 4:** Characteristics of patients with recurrence

Characteristics	*n*	%
Total number of patients	11	
Mean age (range), years	56.9 (34–73)	
Histology		
HGSC	5	45.5
Clear	2	18.2
Neuroendocrine carcinoma	2	18.2
Seromucinous	1	9.1
Adenocarcinoma	1	9.1

HGSC, high-grade serous carcinoma

### Characterization

**Fig. 1:**
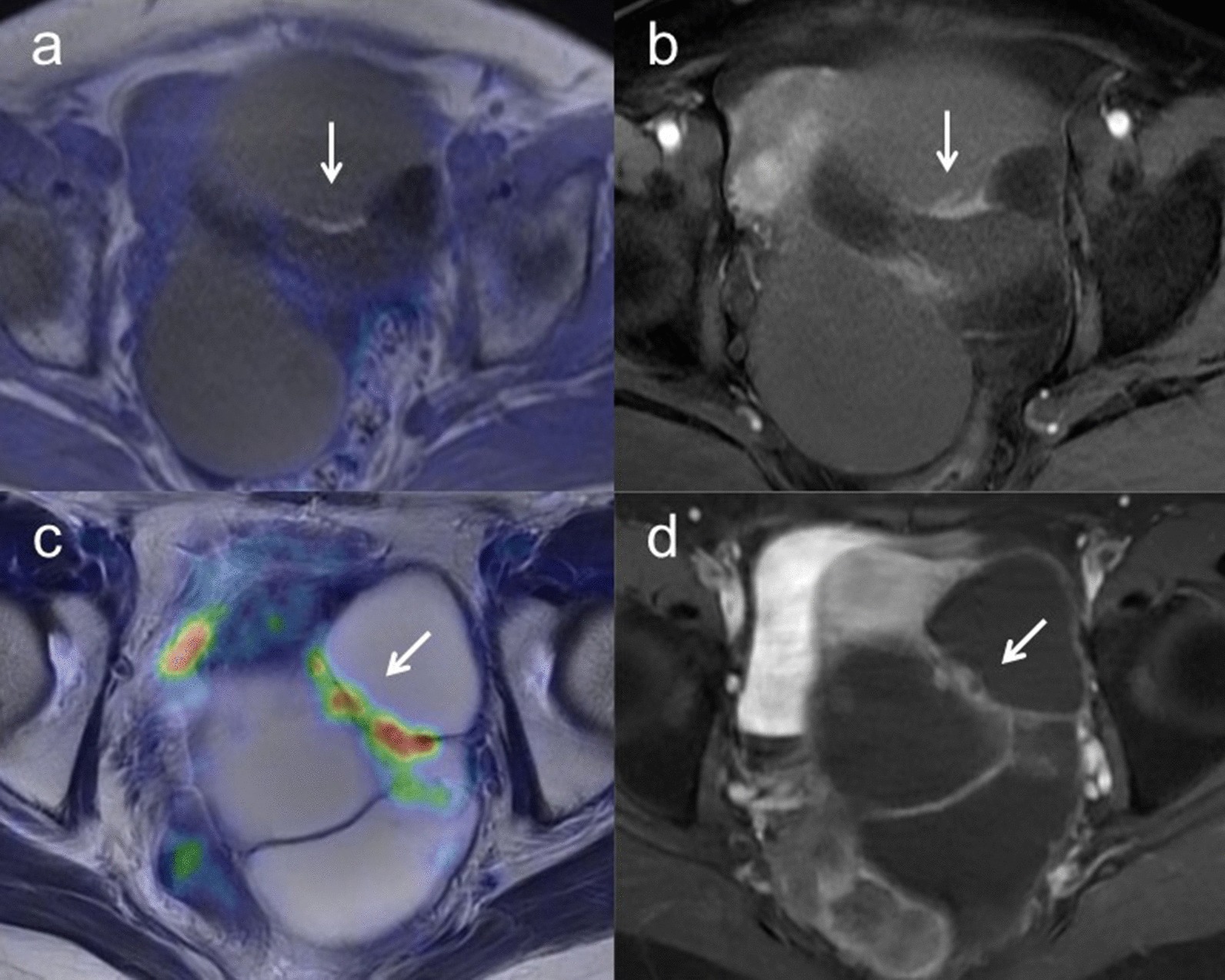
**a** A 57-year-old woman with left ovarian tumor. Axial T2-weighted PET/MR image shows wall thickening without FDG uptake (arrow) in the polycystic left ovarian tumor. **b** Axial T1-weighted contrast-enhanced MR image shows wall thickening with good enhancement (arrow), suggesting the possibility of malignancy. However, histopathologic examination confirmed mucinous cystadenoma without malignancy. **c** A 53-year-old woman with left ovarian tumor. Axial T2-weighted PET/MR image shows wall thickening with FDG uptake (arrow) in the polycystic left ovarian tumor. **d** Axial T1-weighted contrast-enhanced MR image shows wall thickening with good enhancement (arrow). These findings strongly suggest potential malignancy and histopathologic examination confirmed seromucinous borderline tumor

Sensitivity, specificity and accuracy for characterization were 97.4% [95% confidence interval (CI) 0.90–1.00], 86.2% (95% CI 0.77–0.89) and 92.5% (95% CI 0.84–0.95) for [^18^F]FDG PET/MRI and 97.4% (95% CI 0.89–1.00), 58.6% (95% CI 0.48–0.61) and 80.6% (95% CI 0.72–0.83) for ceMRI, respectively (*p* = 0.01) (Table 5). Figure 1 shows representative images for characterizations.

**Table 5:** Comparison of [^18^F]FDG PET/MRI with ceMRI and/or ceCT for patient-based T, N and M staging, detection of residual disease after neoadjuvant chemotherapy and detection of recurrence

	[^18^F]FDG PET/MRI (95% CI)	ceMRI and ceCT (95% CI)	*P*
Primary tumor			
Sensitivity	97.4% (0.90–1.00) (37/38)	97.4% (0.89–1.00) (37/38)	
Specificity	86.2% (0.77–0.89) (25/29)	58.6% (0.48–0.61) (17/29)	
Accuracy	92.5% (0.84–0.95) (62/67)	80.6% (0.72–0.83) (54/67)	0.01
T staging			
Accuracy	96.4% (0.96–0.96) (54/56)	92.9% (0.93–0.93) (52/56)	0.48
T2a (growth into uterus, fallopian tubes, or ovaries)			
Sensitivity	100% (0.43–1.00) (2/2)	50% (0.11–0.50) (1/2)	
Specificity	100% (0.96–1.00) (30/30)	100% (0.97–1.00) (30/30)	
Accuracy	100% (0.93–1.00) (32/32)	96.9% (0.92–0.97) (31/32)	1.00
T2b (growth into other nearby pelvic organs such as bladder, sigmoid colon, or rectum)			
Sensitivity	0% (0.00–0.00) (0/1)	0% (0.00–0.00) (0/1)	
Specificity	100% (1.00–1.00) (31/31)	100% (1.00–1.00) (31/31)	
Accuracy	96.9% (0.97–0.97) (31/32)	96.9% (0.97–0.97) (31/32)	1.00
T3b (growth into organs outside the pelvis, but ≤ 2 cm across)			
Sensitivity	95.5% (0.86–0.96) (21/22)	90.9% (0.81–0.91) (20/22)	
Specificity	100% (0.94–1.00) (33/33)	100% (0.93–1.00) (33/33)	
Accuracy	98.2% (0.91–0.98) (54/55)	96.4% (0.88–0.96) (53/55)	1.00
T3c (growth into organs outside the pelvis, > 2 cm across)			
Sensitivity	100% (0.92–1.00) (21/21)	100% (0.92–1.00) (21/21)	
Specificity	100% (0.95–1.00) (34/34)	100% (0.95–1.00) (34/34)	
Accuracy	100% (0.94–1.00) (55/55)	100% (0.94–1.00) (55/55)	0.00
N staging			
Sensitivity	100% (0.74–1.00) (6/6)	33.3% (0.12–0.33) (2/6)	
Specificity	100% (0.93–1.00) (21/21)	100% (0.94–1.00) (21/21)	
Accuracy	100% (0.88–1.00) (27/27)	85.2% (0.76–0.85) (23/27)	0.13
M staging			
Sensitivity	100% (0.94–1.00) (12/12)	25.0% (0.19–0.25) (3/12)	
Specificity	100% (0.25–1.00) (1/1)	100% (0.22–1.00) (1/1)	
Accuracy	100% (0.88–1.00) (13/13)	30.8% (0.19–0.31) (4/13)	< 0.01
Evaluation of residual disease for interval debulking surgery after neoadjuvant chemotherapy			
Sensitivity	71.4% (0.71–0.71) (5/7)	57.1% (0.57–0.57) (4/7)	
Specificity	0% (0.00–0.00) (0/0)	0% (0.00–0.00) (0/0)	
Accuracy	71.4% (0.71–0.71) (5/7)	57.1% (0.57–0.57) (4/7)	1.00
Evaluation of recurrence			
Sensitivity	100% (0.88–1.00) (9/9)	88.9% (0.76–0.89) (8/9)	
Specificity	100% (0.44–1.00) (2/2)	100% (0.40–1.00) (2/2)	
Accuracy	100% (0.80–1.00) (11/11)	90.9% (0.69–0.91) (10/11)	1.00

### T staging

**Fig. 2:**
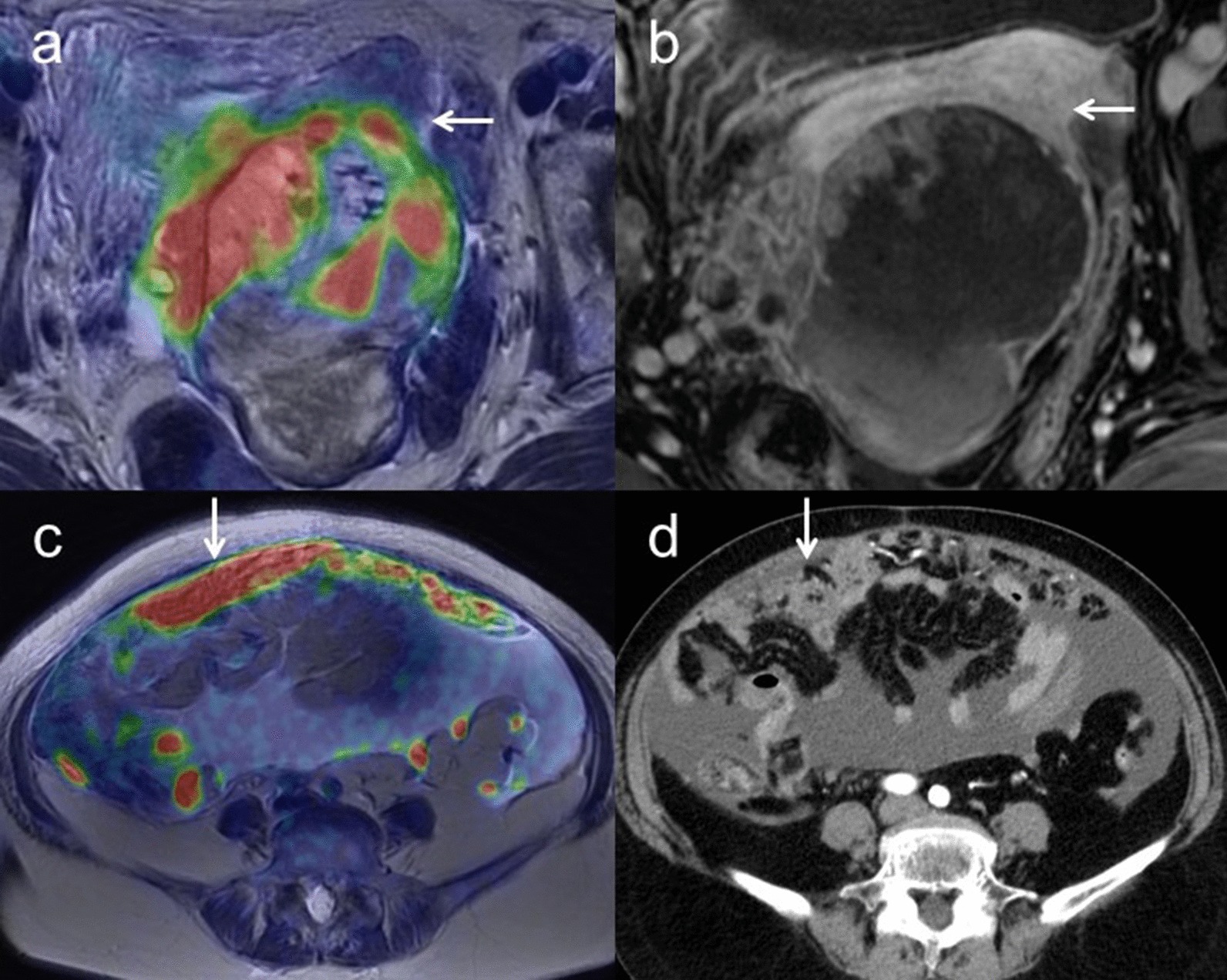
**a** A 67-year-old woman with right ovarian tumor. Axial T2-weighted PET/MR image shows a papillary solid part with FDG uptake invading the posterior uterine myometrium (arrow) in a polycystic right ovarian tumor. **b** Axial T1-weighted contrast-enhanced MR image shows the papillary solid part with good enhancement (arrow) and unclear findings of growth into the uterus. Histopathologic examination confirmed carcinosarcoma with growth into the posterior uterine myometrium (T2a). **c** A 62-year-old woman with suspected ovarian cancer. Axial T2-weighted PET/MR image shows the omental cake with FDG uptake (arrow). **d** Contrast-enhanced CT shows thickening of the omentum with good enhancement (arrow). These findings strongly suggest potential malignancy with carcinomatous peritonitis and histopathologic examination confirmed high-grade serous carcinoma with carcinomatous peritonitis (T3c)

Overall accuracies of T staging for [^18^F]FDG PET/MRI and ceMRI/ceCT were 96.4% (95% CI 0.96–0.96) (54/56) and 92.9% (95% CI 0.93–0.93) (52/56), respectively (*p* = 0.48).[^18^F]FDG PET/MRI understaged the actual T stage in two patients (3.6%), whereas ceMRI/ceCT resulted in understaging in four patients (7.1%).[^18^F]FDG PET/MRI incorrectly classified one T2b and one T3b tumors as T1, whereas ceMRI/ceCT incorrectly classified one T2a, one T2b and two T3b tumors as T1. Sensitivity, specificity and accuracy for detecting growth into the uterus, fallopian tubes or ovaries were 100% (95% CI 0.43–1.00), 100% (95% CI 0.96–1.00) and 100% (95% CI 0.93–1.00) for [^18^F]FDG PET/MRI, and 50% (95% CI 0.11–0.50), 100% (95% CI 0.97–1.00) and 96.9% (95% CI 0.92–0.97) for ceMRI, respectively (*p* = 1.00). Sensitivity, specificity and accuracy for growth into other nearby pelvic organs such as the bladder, sigmoid colon, or rectum were 0% (95% CI 0.00–0.00), 100% (95% CI 1.00–1.00) and 96.9% (95% CI 0.97–0.97) for [^18^F]FDG PET/MRI and 0% (95% CI 0.00–0.00), 100% (95% CI 1.00–1.00) and 96.9% (95% CI 0.97–0.97) for ceMRI, respectively (*p* = 1.00). Sensitivity, specificity and accuracy for growth into organs outside the pelvis and no bigger than 2 cm in extent were 95.5% (95% CI 0.86–0.96), 100% (95% CI 0.94–1.00) and 98.2% (95% CI 0.91–0.98) for [^18^F]FDG PET/MRI and 90.9% (95% CI 0.81–0.91), 100% (95% CI 0.93–1.00) and 96.4% (95% CI 0.88–0.96) for ceCT, respectively (*p* = 1.00). Sensitivity, specificity and accuracy for growth into organs outside the pelvis and larger than 2 cm in extent were 100% (95% CI 0.92–1.00), 100% (95% CI 0.95–1.00) and 100% (95% CI 0.94–1.00) for [^18^F]FDG PET/MRI and 100% (95% CI 0.92–1.00), 100% (95% CI 0.95–1.00) and 100% (95% CI 0.94–1.00) for ceCT, respectively (*p* = 0.00) (Table 5). Figure 2 shows representative images for T2 and T3 staging.

### N staging

**Fig. 3:**
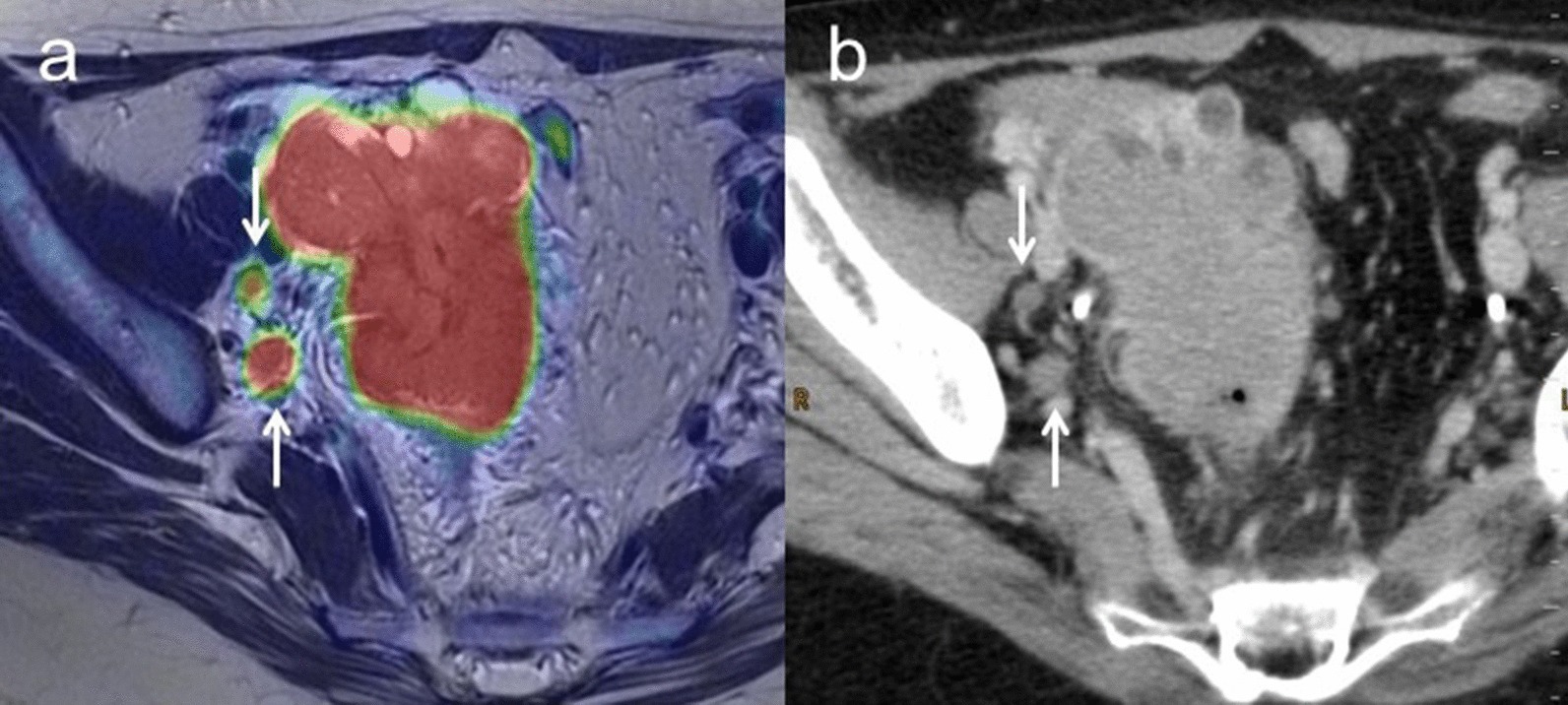
**a** A 50-year-old woman with pathologically confirmed ovarian cancer. Axial T2-weighted PET/MR image shows right pelvic lymph nodes with FDG uptake (arrow). **b** Contrast-enhanced CT shows right pelvic lymph nodes less than 1 cm in short-axis diameter without enhancement (arrow). After NAC, these lymph nodes are decreased in size and SUV, suggesting these nodes as a sign of malignancy (N1)

Patient-based sensitivity, specificity and accuracy for N staging including retroperitoneal lymph nodes metastasis were 100% (95% CI 0.74–1.00), 100% (95% CI 0.93–1.00) and 100% (95% CI 0.88–1.00) for [^18^F]FDG PET/MRI and 33.3% (95% CI 0.12–0.33), 100% (95% CI 0.94–1.00) and 85.2% (95% CI 0.76–0.85) for ceCT, respectively (*p* = 0.13). ceCT incorrectly classified four N1 lymph nodes as N0 (Table 5). Lymph node metastasis was confirmed histologically in one case, whereas we regarded these lymph nodes as a sign of malignancy because of a decrease in size and/or SUVmax under NAC in the remaining three cases. Lesion-based sensitivity, specificity and accuracy for N staging including retroperitoneal lymph nodes metastasis were 78.6% (95% CI 0.57–0.91), 95.7% (95% CI 0.93–0.97) and 93.9% (95% CI 0.89–0.97) for [^18^F]FDG PET/MRI, and 42.9% (95% CI 0.24–0.58), 96.6% (95% CI 0.94–0.98) and 90.8% (95% CI 0.87–0.94) for ceCT, respectively. Sensitivity showed a tendency toward a difference (*p* = 0.07), and specificity and accuracy were not significant (*p* = 1.00 and *p* = 0.29, respectively) (Table 6). Figure 3 shows representative images for N staging.

**Table 6:** Comparison of [^18^F]FDG PET/MRI and ceCT for lesion-based nodal metastasis

	[^18^F]FDG PET/MRI (95% CI)	ceCT (95% CI)	*P*
Sensitivity	78.6% (0.57–0.91) (11/14)	42.9% (0.24–0.58) (6/14)	0.07
Specificity	95.7% (0.93–0.97) (112/117)	96.6% (0.94–0.98) (113/117)	1
Accuracy	93.9% (0.89–0.97) (123/131)	90.8% (0.87–0.94) (119/131)	0.29

### M staging

**Fig. 4:**
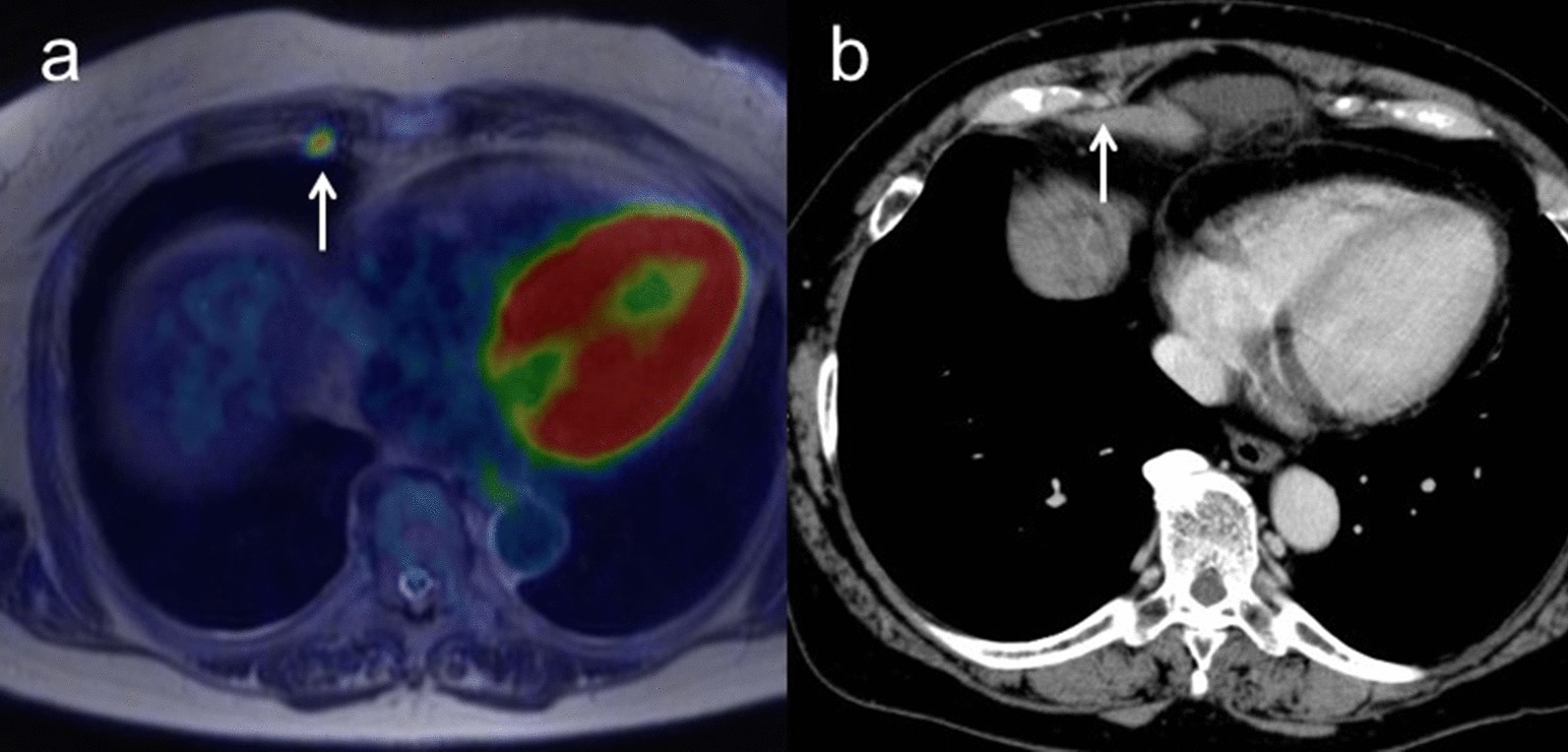
**a** A 66-year-old woman with pathologically confirmed ovarian cancer. Axial T2-weighted PET/MR image shows a right parasternal lymph node with FDG uptake (arrow). **b** Contrast-enhanced CT shows a right parasternal lymph node less than 1 cm in short-axis diameter with slightly enhancement (arrow). After NAC, this lymph node decreased in size and SUV, suggesting that this node as a sign of malignancy (M1)

Sensitivity, specificity and accuracy for M staging were 100% (95% CI 0.94–1.00), 100% (95% CI 0.25–1.00) and 100% (95% CI 0.88–1.00) for [^18^F]FDG PET/MRI, and 25.0% (95% CI 0.19–0.25), 100% (95% CI 0.22–1.00) and 30.8% (95% CI 0.19–0.31) for ceCT, respectively (*p* < 0.01). ceCT incorrectly classified nine M1 tumors as M0 (Table 5). Figure 4 shows representative images for M staging.

### Residual disease for IDS after NAC

**Fig. 5:**
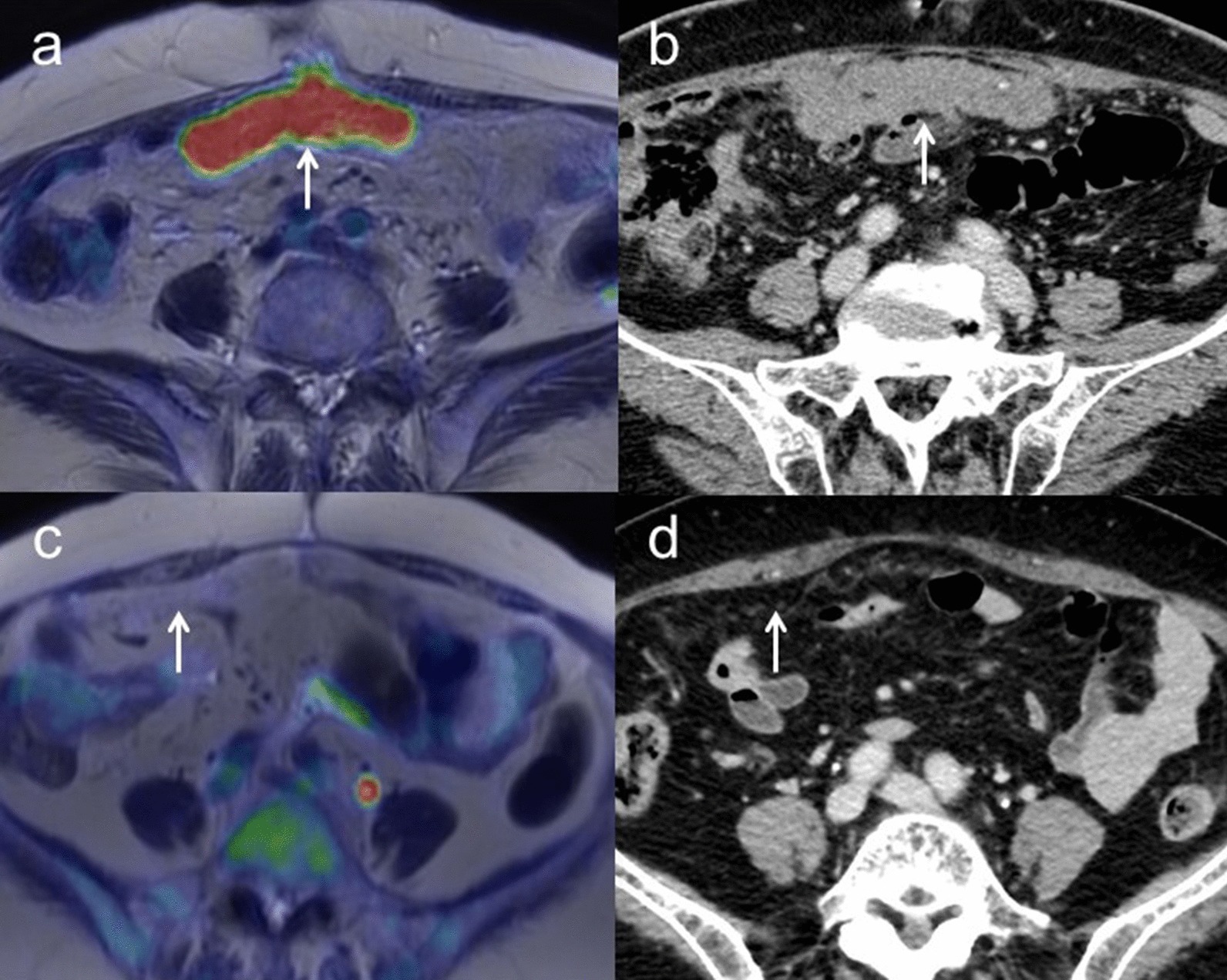
**a** A 71-year-old woman with ovarian cancer showing carcinomatous peritonitis and pleural effusion confirming the presence of malignant cells. After NAC, axial T2-weighted PET/MR image shows the omental cake with FDG uptake (arrow). **b** Contrast-enhanced CT shows the thickened omentum with slight enhancement (arrow). These findings strongly suggested potential residual disease, which was confirmed by histopathologic examination. **c** A 77-year-old woman with ovarian cancer with carcinomatous peritonitis and ascites confirming the presence of malignant cells. After NAC, axial T2-weighted PET/MR image shows the almost disappear of the omental cake and FDG uptake (arrow). **d** Contrast-enhanced CT also shows the almost disappear of thickness and enhancement of the omentum (arrow). These findings show the marked response to NAC. However, histopathologic examination confirmed residual disease comprising high-grade serous carcinoma with carcinomatous peritonitis

Sensitivity, specificity and accuracy for detecting residual disease for IDS after NAC were 71.4% (95% CI 0.71–0.71), 0% (95% CI 0.00–0.00) and 71.4% (95% CI 0.71–0.71) for [^18^F]FDG PET/MRI, and 57.1% (95% CI 0.57–0.57), 0% (95% CI 0.00–0.00) and 57.1% (95% CI 0.57–0.57) for ceCT, respectively (*p* = 1.00) (Table 5). Figure 5 shows representative images for detecting residual disease for IDS after NAC.

### Recurrence

**Fig. 6:**
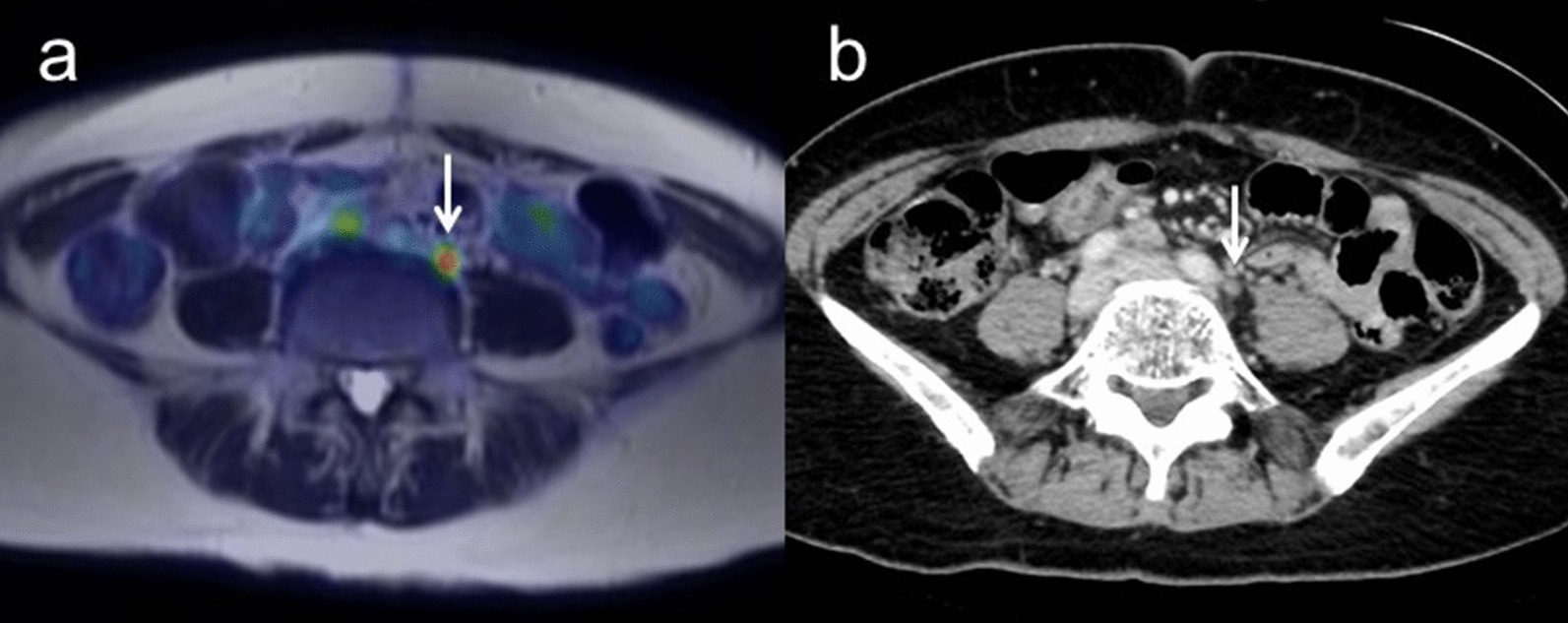
**a** A 63-year-old woman with pathologically confirmed ovarian cancer. During follow-up after the successful initial treatment, including surgery and first-line chemotherapy, CA-125 gradually increased. Axial T2-weighted PET/MR image shows the left para-aortic lymph node with FDG uptake (arrow). **b** Contrast-enhanced CT shows the left para-aortic lymph node less than 1 cm in short-axis diameter with slightly enhancement (arrow). After second-line chemotherapy, this lymph node decreased in size and SUV, suggesting this node as a sign of recurrence

Sensitivity, specificity and accuracy for detecting recurrence were 100% (95% CI 0.88–1.00), 100% (95% CI 0.44–1.00) and 100% (95% CI 0.80–1.00) for [^18^F]FDG PET/MRI, and 88.9% (95% CI 0.76–0.89), 100% (95% CI 0.40–1.00) and 90.9% (95% CI 0.69–0.91) for ceCT, respectively (*p* = 1.00) (Table 5). Figure 6 shows representative images for recurrence.

## Discussion

To the best of our knowledge, this is the first study to investigate the diagnostic value of non-contrast [^18^F]FDG PET/MRI for ovarian cancer in comparison with conventional imaging modalities such as ceMRI and ceCT. For the characterization of ovarian tumor and M staging, [^18^F]FDG PET/MRI offered significantly superior accuracy to ceMRI and ceCT, while the accuracies of [^18^F]FDG PET/MRI were equivalent to those of ceMRI and ceCT for T and N staging, detection of residual disease for IDS after NAC and detection of recurrence. These findings suggest that [^18^F]FDG PET/MRI might provide a useful alternative to conventional imaging modalities in ovarian cancer.

Most ovarian tumors are detected incidentally or clinically, and differentiation between benign or malignant disease is important to optimize decision-making for surgical options such as laparoscopic surgery or staging laparotomy in the treatment of ovarian tumor. MRI constitutes the gold standard for the characterization of suspected ovarian malignancy, and MRI with intravenous contrast administration provides the highest post-test probability of detecting ovarian cancer, compared with CT, US with color Doppler or MRI without contrast administration [3]. The utility of [^18^F]FDG PET/CT in the differentiation of benign and malignant ovarian tumors is reportedly limited because of false-positives resulting from physiological conditions such as inflammation or the menstrual cycle and false-negatives in diagnosing early stage or borderline malignancies [11]. In terms of [^18^F]FDG PET/MRI, only fused [^18^F]FDG PET/MRI has been reported and showed higher sensitivity of 94% and specificity of 100% for the characterization of ovarian tumors compared with MRI and [^18^F]FDG PET/CT [14]. In the present study, pelvic PET/MRI was performed as a delayed PET scan with high-resolution MR images, allowing accurate discrimination of hypermetabolic malignancies from benign FDG uptake of benign tumors and inflammation, resulting in higher accuracy [92.5% (95% CI 0.84–0.95)] compared to ceMRI [80.6% (95% CI 0.72–0.83)]. Application of [^18^F]FDG PET/MRI including delayed regional PET and high-resolution MR scans could provide higher diagnostic value than conventional modalities for characterizing ovarian tumor. Moreover, PET/MRI may be superior to PET/CT, particularly for imaging gynecologic tumors due to the excellent soft-tissue contrast, leading to accurate identification of small hypermetabolic malignancies from the adjacent organs with physiological metabolic activity such as the bladder, ureter, or intestines. Recently, introduced PET systems using silicon photomultipliers with digital readout (dPET) have been reported to offer improved timing and spatial resolution over conventional PET systems, leading to the detection of small lesions and accurate staging in some cancers such as lung or breast cancer [17, 18]. Although comparative studies with dPET and conventional PET combined with MRI are needed in the future, introduction of the dPET to PET/MRI may also improve the diagnostic accuracy for characterizing ovarian tumors.

The spread of ovarian cancer into adjacent organs such as the uterus, sigmoid colon, bladder and rectum may be better appreciated on MRI than on CT in ovarian cancer, as seen for other gynecologic cancers [19–21]. In terms of [^18^F]FDG PET/CT, limited data are available regarding the assessment of extension into adjacent organs in gynecologic cancers. The results of [^18^F]FDG PET/CT were comparable to those of ceMRI and transvaginal US for predicting myometrial invasion and were superior to results from those modalities for identifying cervical invasion in endometrial cancer [22], while [^18^F]FDG PET/CT has been reported to offer lower accuracy than MRI in the assessment of vaginal and parametrial invasion in cervical cancer [23]. This suggests that the utility of [^18^F]FDG PET/CT in assessing local extension into the adjacent organs is still controversial. Recently, the diagnostic potential of integrated PET/MRI has been reported in these situations.[^18^F]FDG PET/MRI correctly identified T stage (85%) in cervical cancer [24], suggesting that [^18^F]FDG PET/MRI might be useful in the local evaluation of the ovarian cancer. In the assessment of T3b and T3c staging, ceCT has been widely used as the standard imaging modality for staging ovarian cancer. When detecting peritoneal disease, multidetector-row CT, MRI and [^18^F]FDG PET/CT offered sensitivities of 96%, 98% and 95% and specificities of 92%, 84% and 96%, respectively, showing no significant differences [25]. In terms of [^18^F]FDG PET/MRI, abdominal metastasis including peritoneal was accurately detected as well as with [^18^F]FDG PET/CT [13]. The present study showed that the accuracy of [^18^F]FDG PET/MRI for T staging, including local evaluation and detection of peritoneal disease, was equivalent to that of ceMRI and ceCT, suggesting that [^18^F]FDG PET/MRI might not provide additional value over ceMRI or ceCT in the T staging of ovarian cancer.

N and M staging have also been performed using ceCT. For the detection of retroperitoneal lymph node metastasis, [^18^F]FDG PET/CT offered greater accuracy compared with CT and MRI, while no significant difference was evident among them [8]. Moreover, [^18^F]FDG PET/CT could improve lesion-based accuracy compared with CT, and allow the detection of unpredicted extra-abdominal lymph node metastases or concomitant malignant tumors, suggesting that [^18^F]FDG PET/CT could offer suitable diagnostic performance in detecting distant metastasis, such as that in the mediastinal or supraclavicular lymph nodes [26]. In terms of [^18^F]FDG PET/MRI, no reports appear to have described the assessment of N and M staging for ovarian cancer. In other cancers, including nasopharyngeal, breast and colorectal cancers, [^18^F]FDG PET/MRI has been reported as superior compared with [^18^F]FDG PET/CT for detecting lymph nodes or distant metastasis [27]. Moreover, [^18^F]FDG PET/MRI has been reported as equivalent to MRI in cervical cancer [24] for the detection of lymph nodes or distant metastasis. Considering the results of the present study, which showed the superiority of [^18^F]FDG PET/MRI to ceCT for M staging, [^18^F]FDG PET/MRI might offer a useful alternative imaging modality to ceCT, or [^18^F]FDG PET/CT in the assessment of N and M staging as well as in the T staging in ovarian cancer. We could not show the superiority of [^18^F]FDG PET/MRI over ceCT for N staging, although previous reports have suggested the superiority of [^18^F]FDG PET/MRI compared with conventional modalities in other cancers [27]. A possible reason for this lack of difference could be the small number of events and samples in our study. Further studies with larger sample sizes are warranted to elucidate the diagnostic value for the detection of lymph node metastasis. In terms of detecting distant metastasis, [^18^F]FDG PET/MRI offered better sensitivity than ceCT. Both [^18^F]FDG PET/MRI and ceCT could detect all liver lesions, whereas only [^18^F]FDG PET/MRI detected all extra-abdominal lymph nodes metastases. Patients with discrepant staging results from these imaging modalities received NAC followed by IDS after the disappearance of metabolic activity from distant metastatic lesions instead of PDS, suggesting that [^18^F]FDG PET/MRI might enable improved treatment planning in such patients.

In terms of predicting treatment response, particularly for NAC, the concentration of CA-125 before and after the third course of NAC could provide an independent predictor for completion of IDS [28]. In terms of [^18^F]FDG PET/CT, reductions in SUV before and after the third to fourth courses of NAC could be associated with histopathological response and may allow differentiation between responders and non-responders [29]. No reports appear to have described [^18^F]FDG PET/MRI for ovarian cancer. In cervical cancer, [^18^F]FDG PET/MRI in pre- and post-treatment examinations could differentiate between radiotherapy responders and non-responders [30]. This suggests that [^18^F]FDG PET/MRI might be useful to identify NAC responders or non-responders in ovarian cancer. However, our results did not show superiority over ceCT for detecting residual disease after NAC for IDS, because some patients had micro-metastasis or peritoneal carcinomatosis less than a few millimeters that could not be detected on [^18^F]FDG PET/MRI.

With epithelial ovarian cancer, more than half of patients experience disease recurrence within two years, irrespective of the effectiveness of first-line chemotherapy. Early detection of recurrence can help in planning optimal treatment, including chemotherapy, radiation or secondary cytoreductive surgery. CA-125 has often been used in monitoring to detect recurrent disease in cases of initially high CA-125, although the National Comprehensive Cancer Network guidelines recommend delaying treatment until clinical evidence indicates relapse [31]. In a meta-analysis of diagnostic accuracy for detecting recurrent disease with CA-125, [^18^F]FDG PET/CT, CT and MRI, [^18^F]FDG PET/CT offered the highest sensitivity of 91%, compared with 69% for CA-125, 79% for CT and 75% for MRI, suggesting that [^18^F]FDG PET/CT could be a useful tool for detecting recurrence, particularly in patients with increased CA-125 and negative CT or MRI [9]. In terms of [^18^F]FDG PET/MRI, a meta-analysis showed that [^18^F]FDG PET/MRI provides excellent diagnostic performance with 96% sensitivity and 95% specificity for restaging female patients with suspected recurrence of gynecological pelvic malignancies [12]. Although no significant differences were identified in comparisons with ceCT in the present study, likely because of the small sample size, [^18^F]FDG PET/MRI could detect recurrent lesions in all patients, suggesting that [^18^F]FDG PET/MRI might also prove useful for detecting recurrent ovarian cancer.

This study had several limitations. First, this investigation used a retrospective design, and not all MRI examinations were performed at our institution. However, our readers re-evaluated the images from other hospitals and were blinded to the initial imaging findings. Second, the sample size of this study was small, particularly in the detection of residual disease for IDS after NAC and detection of recurrence. In these situations, further studies with larger sample sizes are needed to clarify the diagnostic performance of [^18^F]FDG PET/MRI. Third, we could not evaluate histopathological correlations with imaging in patients who had not yet undergone lymphadenectomy. We thus performed node-specific comparisons between imaging and histopathology in all other patients. Fourth, the population included was very heterogeneous and the results of diagnostic performance may reflect different clinical settings. However, we included all patients with suspected ovarian cancer between February 2016 and May 2019, suggesting that this study population may better reflect clinical situations, where preoperatively distinguishing pathologies is often difficult using imaging modalities, particularly for malignant surface epithelial–stromal tumors, sex cord stromal tumors and germ cell tumors [32].

## Conclusion

[^18^F]FDG PET/MRI combines the individual advantages of PET and MRI for whole-body and detailed regional scans, and could provide additional value when the classification of a malignant or benign ovarian tumor is in doubt. Moreover, [^18^F]FDG PET/MRI offers better sensitivity for detecting distant metastasis than ceCT, suggesting that this modality might enable improved treatment planning.

## Supplementary information

**Additional file1:** Characteristics of patients excluded from the present study.

## Data Availability

The datasets used and/or analyzed during the current study are available from the corresponding author on reasonable request.
