# Supplementary material for: Diagnostic value of [18F]FDG PET/MRI for staging in patients with ovarian cancer
Source: EJNMMI Res. 2020 Oct 2;10:117. doi: 10.1186/s13550-020-00712-3 (PMC7532239; doi:10.1186/s13550-020-00712-3)
Supplement: Supplementary file 1 — Additional file1: Characteristics of patients excluded from the present study. [file 13550_2020_712_MOESM1_ESM.doc]

Additional file 1

Table S1. Characteristics of patients excluded from the present study

| Characteristics | n | % |
| --- | --- | --- |
| Total number of patients | 32 |  |
| Histology |  |  |
| HGSC | 7 | 21.9 |
| mucinous carcinoma | 1 | 3.1 |
| clear | 1 | 3.1 |
| adenocarcinoma | 2 | 6.2 |
| serous cystadenoma | 4 | 12.5 |
| mucinous cystadenoma | 6 | 18.8 |
| endometrial cyst | 4 | 12.5 |
| mature cystic teratoma | 3 | 9.4 |
| fibroma | 4 | 12.5 |

HGSC, high-grade serous carcinoma
